# Self-Learning Power Control in Wireless Sensor Networks

**DOI:** 10.3390/s18020375

**Published:** 2018-01-27

**Authors:** Michele Chincoli, Antonio Liotta

**Affiliations:** 1 Electrical Engineering Department, Eindhoven University of Technology, De Zaale (internal address: Groene Loper 19), 5612 AJ Eindhoven, The Netherlands; 2 Data Science Centre, University of Derby, Lonsdale House, Quaker Way, Derby DE1 3HD, UK; a.liotta@derby.ac.uk

**Keywords:** wireless sensor network, transmission power control, Q-learning, reinforcement learning, game theory, multi-agent, energy efficiency, quality of service

## Abstract

Current trends in interconnecting myriad smart objects to monetize on Internet of Things applications have led to high-density communications in wireless sensor networks. This aggravates the already over-congested unlicensed radio bands, calling for new mechanisms to improve spectrum management and energy efficiency, such as transmission power control. Existing protocols are based on simplistic heuristics that often approach interference problems (i.e., packet loss, delay and energy waste) by increasing power, leading to detrimental results. The scope of this work is to investigate how machine learning may be used to bring wireless nodes to the lowest possible transmission power level and, in turn, to respect the quality requirements of the overall network. Lowering transmission power has benefits in terms of both energy consumption and interference. We propose a protocol of transmission power control through a reinforcement learning process that we have set in a multi-agent system. The agents are independent learners using the same exploration strategy and reward structure, leading to an overall cooperative network. The simulation results show that the system converges to an equilibrium where each node transmits at the minimum power while respecting high packet reception ratio constraints. Consequently, the system benefits from low energy consumption and packet delay.

## 1. Introduction

Wireless Sensor Networks (WSNs) are dense mesh networks, thus techniques of multiple access to the medium are necessary to manage the communications among the nodes [1]. Such techniques suggest multiple transmissions from different sources, which should not interfere with one another. The transmissions can be scheduled in various dimensions. For instance, in the case of Time Division Multiple Access (TDMA), the time is divided in slots, which are assigned to each source for transmission. Only one stream of information can occur per slot, so multiple transmissions are serialized. In Frequency Division Multiple Access (FDMA), the traffic of the sources are parallelized in frequency per allocated channel. Simultaneous communications can happen, proportionally to the number of channels available. In Code Division Multiple Access (CDMA), transmitters can send packets at the same time, using orthogonal codes multiplied to the symbols that are transmitted/received. In this case, the number of simultaneous transmissions is limited to the number of orthogonal codes. In addition, two more techniques allow concurrent transmissions: Space Division Multiple Access (SDMA) and Carrier Sense Multiple Access (CSMA). These techniques are very sensitive to the dynamics of the network. In SDMA, the communications are confined in zones of the network to reduce contention, while maintaining the connectivity of the network. Using CSMA, the spectrum is scanned and analysed to discover the interested carrier. Both SDMA and CSMA are regulated by either a standard or a de facto protocol. Their advantage is that communications are flexible and decentralized, without the need of synchronization as in TDMA and FDMA. The drawback is that they do not ensure a network free of collision. Thus, the design of the regulations is important for the minimization of the collisions.

Spatial reuse and contention mitigation in SDMA are obtained by dimming the transmission power of the nodes in a network. The decision of the transmission power is dependent on many factors: distance between transmitter and receiver, density of the network, energy available by the nodes, fading of the channel, mobility and performance constraints. In particular, two fields of protocols can be considered for transmission power decision, namely Topology Control (TC) and Transmission Power Control (TPC) [2]. The former acts at the network layer, thus the value of transmission power per node is chosen with respect to the routing path that the traffic can take to reach the destination. The path can either be point-to-point or multi-hop with variable number of hops. Unlike TC, TPC is link-based, used only for point-to-point communications by means of link quality monitoring.

TPC is used in WSNs, whereby energy and spectrum efficiency are particularly critical. Energy consumption is a typical problem, given the constrained resources of the sensor nodes, including limited battery capacity [3]. The latter should guarantee a long period of lifetime, more than a decade, where the sensor nodes operate without human intervention [4]. For this reason, sleeping techniques for energy efficiency are proposed in the literature to carefully allocate operational time and reduce idle periods [5,6].

WSNs were initially developed as independent, isolated entities for Machine to Machine (M2M) communications, providing simple operations, such as the monitoring of physical phenomena and executing actions. Therefore, the energy efficiency was prioritized by researchers, neglecting the Quality of Service (QoS). Instead, in the context of Internet of Things (IoT), WSNs are interconnected with other networks, providing services to users through Internet (e.g., smart meters [7]). Hence, QoS becomes more relevant, with regard to network reliability, to respect performance requirements of various applications [8].

TPC is a widely studied topic [9], but all the solutions provided up to now are not sufficient for predicting the link quality required to adapt the transmission power to any environment [10,11]. In literature, most of the protocols can be categorized in proactive and reactive classes [12]. In the former case, they use algorithms that are either based on empirical studies or analytical models [13,14,15,16]. In empirical studies, the devices evaluate the link quality of the wireless channel, which is used as a metric for the transmission power selection. The drawback in this approach is that unexpected changes in the environment are not considered, thus the nodes are unprepared and may take wrong actions, sacrificing the network performance. Instead, the algorithms based on analytical models are founded on theoretical basis. In the reactive case, the link quality is continuously monitored and compared with a threshold to adjust the transmission power, adapting to changes in the environment only after these have been sensed [17,18,19,20]. Past events are not accounted for and the result is that transmission power is left to oscillate due to the variability of the channel conditions. This is particularly so, because the link quality varies often in space and time.

To overcome the disadvantages of the proactive and reactive techniques, machine learning represents an attractive solution [21] to reach a defined goal by learning the dynamics of the WSNs [22,23], predicting and adapting the transmission power values in different conditions. The objective is making WSNs autonomous without the intervention of developers and users to set the transmission power. To the best of our knowledge, only few contributions have applied machine learning in TPC, mainly Reinforcement Learning (RL) and fuzzy logic [12]. RL in WSNs has been used in literature but mainly for path selection in routing protocols and sleeping techniques, maintaining constant learning factors [24,25,26]. Static values would either bring the system slowly to convergence or make the system too reactive if the learning factor is constantly low or high respectively. In parallel, a wrong calibration of the explorative policy influences again the speed of convergence and the optimality of the reached value.

Given the density of WSNs and the unreliability of the wireless channel, a centralized approach would be inefficient and resource consuming, with waste of bandwidth for the transfer of information to a central node, risk of packet loss, long packet delay and energy consumption for the nodes involved in the routing process [27]. For this reason, we are interested in developing a distributed TPC protocol. In such a context, the nodes have to coordinate among each other to efficiently allocate the resources [28,29]. Otherwise, if hundreds of smart objects compete for wireless spectrum, it is unimaginable for them to employ aggressive and power-raising policies in face of channel congestion [30]. Nodes should rather cooperate with one another, which requires them to strive for minimum transmission power. In a cooperative approach, every node tunes its transmission power towards a global goal (e.g., interference mitigation and energy savings by transmission power minimization) [31].

In this paper, we propose a smart protocol, as the result of a RL algorithm (i.e., Q-learning) and TPC integration. The protocol is implemented in both the NS3 network simulator and Contiki OS for sensor devices. In NS3, we test the protocol in two scenarios: single agent and multi-agent WSNs, where one agent is the transmitter in a point-to-point communication. For each scenario, the traffic load and the distance between one transmitter and its receiver vary. A comparison is performed with Homogeneous networks (HG), in which nodes transmit at one constant power level, considering all available power levels. In each case, network performance is evaluated in terms of Packet Reception Ratio (PRR), packet delay and energy per bit. The comparison is meant to show the benefits of the learning protocol with respect to choosing an arbitrary transmission power. HG is also considered the reference for the performance of the network, indicating the transmission power levels at which the nodes should learn to set.

This paper shows that for different network conditions, there exists a trade-off among the performance obtained using different transmission power levels. For instance, we see that using high transmission power in the homogeneous network, PRR and latency either stay constant or are only improved by a decimal factor with respect to transmitting at lower power levels, thus extra energy is wasted. However, using low transmission power, in network conditions of detrimental path loss and interference, the nodes have the counter-effect of consuming more energy and performing worse than at higher power because of retransmissions. Through our protocol, we discover a near-optimal equilibrium in the system that provides a balance between reliability, in the sense of packet reception, and energy efficiency.

To summarise, we explore the principles of cooperation, distribution and machine learning applied to WSNs. Our contributions are:

- Investigate Q-learning for TPC and analysis of the algorithm convergence by varying the learning factors in time.
- RL based on a Decentralized Partially Observable Markov Decision Process (Dec-POMDP): To the best of our knowledge, this is the first work that applies such method to TPC in WSNs [32]. The nodes of the network learn from past observations by memorizing only the last values of the observed parameters, by using a Markov Decision Process (MDP). Each node is independent and relies on its own local information. Therefore, the system is decentralized and partially observable. To this end, the bandwidth is not spent for the exchange of information with a central node for the purpose of handling a network protocol.
- Indirect collaboration among the devices without the exchange of information: This is possible through the application of the common interest game in Game Theory. The agents in RL are also the players of the game, cooperating towards a common goal that leads to a global benefit by minimizing the transmission power.
- Design and implementation of new modules in NS3 related to TPC. New modules are generated and linked to the physical and MAC layers modules, which are already included in the NS3 open source release. We provide more realistic and reliable results, when compared with other tools used in the literature [33,34,35,36], since NS3 offers the opportunity to analyse many aspects involved in the wireless communications.
- Commercial applicability: To the best of our knowledge, it is the first time that a smart protocol is implemented in sensor devices for TPC. The design of our protocol takes into consideration the industrial application, aiming at putting in practice the concept of autonomic networks. Our protocol is lightweight and suitable for constrained devices having limited memory, processing and energy capabilities. A section of this paper is dedicated to the implementation of our protocol in Contiki OS, for real-world sensor devices, experiments and the discussion of the results.

The remaining part of the paper proceeds as follows: Section 2 provides an overview of reinforcement learning. In Section 3, we identify the works related to our research. Section 4 introduces the Q-learning algorithm, the approach to single-agent and multi-agent systems, the detailed implementation of Q-learning and its integration in a TPC protocol. The simulation setup and the results are explained in Section 5. The experimental setup and results follow in Section 6. Finally, the paper ends with discussions in Section 7 and conclusions and future works in Section 8.

## 2. Background

RL is one of the three big groups in Machine Learning together with supervised learning and unsupervised learning. In supervised learning, the system learns from labelled data, providing a map between input and known output in order to make associations for future data. In unsupervised learning, the data is unlabelled and the system learns how to classify it. Each class is defined by features that help the system to distinguish coming data. Instead, RL is used for self-learning systems in an unknown environment. The training is done either on a batch of stored data [37] or online, using real-time data [38]. In both manners, the data is obtained through observations, taking certain actions. Our protocol lies in the group of RL. Initially the devices do not have information about the environment, thus they adapt to any kind of scenario, learning through real-time data.

RL is a solution to an MDP, whenever the environment respects the Markovian properties. The MDP is a framework of sequential tasks in instants of time, which are required to make decisions. Solutions of MDPs are obtained through different methods that depend on the time horizon of the system. If the system is operative only for a fixed period, the time horizon is finite and the MDPs can be solved by the Bellman optimality equation using either the dynamic programming approach or the value iteration approach. Otherwise, if the system is operative on an infinite time horizon, the solution methods are: value iteration, policy iteration, linear programming, approximation method and online learning [32]. The latter includes RL.

**Figure 1:**
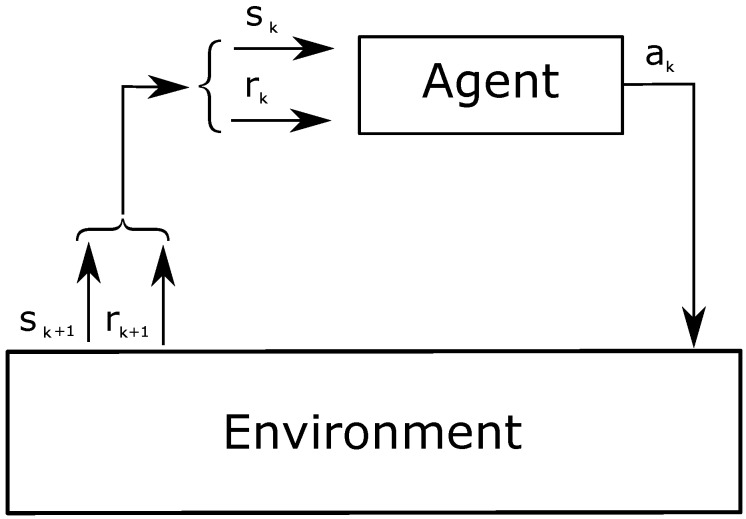
Single agent reinforcement learning schema.

RL involves two main entities: an agent and the environment (Figure 1). The agent is the learner and decision-maker, while the environment is an unknown entity that influences the agent’s performance. The system strives to achieve a specific target; thus, the agent iteratively learns the actions to pursue such target, adapting to various circumstances. Particular attention is required for the definition of the agent and the environment, based on the target. For instance, the environment can be represented as anything that the agent cannot control. The latter observes the environment on an episodic basis, where the episodes are defined by the developer. At a given *k*-th episode, the status of the environment is represented by the value $s_{k}\in S\subseteq N$, where *S* is a set of states. The agent interacts with the environment by taking decisions, called actions $a_{k}\in A\subseteq N$, where *A* is a set of actions. The succession of state–action–state is formulated by transition probabilities. The system decides to select an action under a certain state following a policy, $\pi_{k}\left(a|s\right)$. Actions can have either a positive or negative impact on the system in order to accomplish the target. Thus, the system provides a feedback or reward $r_{k}\in R\subseteq R$ to evaluate the effect of the actions, where *R* is a set of rewards. The reward is both a quality value and the goal of the agent.

Given the fundamental parameters of the system, the Markovian property is defined as follows:

(1) $$\begin{array}{c} Pr\{R_{k+1}=r,S_{k+1}=s|S_{0},A_{0},R_{1},\ldots ,S_{k-1},A_{k-1},R_{k},S_{k},A_{k}\}=\\ Pr\{R_{k+1}=r,S_{k+1}=s|S_{k},A_{k}\} \end{array}$$

Only the last state and action are necessary to know the probability of getting certain states and rewards in the next episode. Hence, it is not necessary to memorize all the past values but only the ones that happened in the last event.

The total reward in the long term, or return of the system, knowing all the values obtained per episode, is calculated by a sum of rewards. To avoid the divergence of the series, the return is multiplied with the discount factor, 0≤γ≤1, obtaining the discounted return *D*:

(2) $$\begin{array}{c} D_{k}=\sum_{n=0}^{\infty }\gamma^{n}\cdot R_{k+n+1} \end{array}$$

The discounted return can be calculated only after getting the rewards. Therefore, to predict the return in advance, we have to calculate the expectation of *D* knowing the current state and action, which is called the action-value function, $q_{\pi }$, under the policy π:

(3) $$\begin{array}{c} q_{\pi }\left(s,a\right)=E_{\pi }\left[D_{k}|S_{k}=s,A_{k}=a\right] \end{array}$$

The target of the system is to maximize the action-value function, thus the agent has to define the optimal policy, $\pi^{*}$, and solve the Bellman optimality equation, as follows:

(4) $$\begin{array}{c} q_{\pi^{*}}\left(s,a\right)=E\left[R_{k+1}+\gamma \cdot \max_{a^{\prime }}q_{\pi^{*}}\left(S_{k+1},a^{\prime }\right)|S_{k}=s,A_{k}=a\right], \end{array}$$

given that $\pi^{*}\left(s\right)=\arg\;\max_{a}q_{\pi^{*}}\left(s,a\right)$ and $q_{\pi^{*}}=\max_{\pi }q_{\pi }$, ∀s∈S. Maximal action values are discovered by pursuing an additive strategy, such as ϵ-greedy, ϵ-soft and softmax.

In this section, we have described RL based on MDP, expressed by the interaction between one agent and the environment, which is entirely observable by the agent. However, in some systems, the environment is not always completely observable; therefore, these systems are based on Partially Observable Markov Decision Process (POMDP). In other cases, systems are based on Multi-Agent Markov Decision Process (MMDP), when they are composed by multiple agents that have a global view of the environment and influence it simultaneously in a cooperative matter. In the same situation but with agents that have a partial view of the environment, the extension of MDP is called Decentralized POMPD (Dec-POMPD). Finally, if multiple agents do not cooperate, systems are based on Stochastic Games (SGs) [32].

## 3. Related Work

As explained in the previous section, RL is one of the possible solutions to MDP. Therefore, we firstly provide a deep investigation to all the works that have addressed MDP for power control with offline solutions, and then we focus only on those that have considered RL. Alsheikh et al. categorize works in the literature per MDP extension and application in WSNs [32]. Among the applications considered in their survey, the one that is related to our work is resource and power optimization. Within the group of works under such application class, the methods use the classic MDP, except for few that are based either on SG or POMDP [39,40]. Furthermore, only one approach based on online learning (i.e., RL) [33] is reported, where the difference against the other solutions is that the system does not know the environment.

For instance, Krishnamurthy et al. apply SG to CDMA ALOHA networks for the selection of the Signal Noise Ratio (SNR) threshold, in order to decide whether to transmit or wait in a determined time slot [39]. The nodes know in advance the channel status before transmitting. The results show the variation of the threshold and throughput with respect to the number of nodes in the network. The proposed method is compared with two cases where there is no control and no channel information respectively. Instead, Udenze et al. apply POMDP in their system, which is formed by one transmitter node (i.e., the agent) that interacts with a static environment [40]. The wireless channel is without a fading model, and the interferers have only one power level available for transmission. Since the agent observes partially the environment, it creates belief states as an estimation of the environment status (i.e., the interferers are either idle or transmitting). The actions that the agent can take are either a transmission with low power or high power, wait idle or listen to the channel. The policy to take one action under one belief state is set by fixed transition probabilities. The agent gets either a positive reward, if a packet is received, or a negative one otherwise. The authors show how the computational time varies by increasing the number of states and actions. Other works presented in the survey [32] use MDP [34,35,36,41]. An industrial application is studied by Gatsis et al., where the state information of one plant is transmitted by a sensor node to a controller [34]. The goal is to minimize the transmission power and the state estimation error at the controller side. The optimal power is obtained following an MDP formulation and solving the Bellman equation. However, given the onerous calculations that are needed to solve the Bellman equation, only the expression of the optimal power is provided. Then, the approximate dynamic programming is used to define the suboptimal power policy, which is evaluated through numerical simulations. The results show the transmission decision (transmit or not) with respect to two plant states and the channel gain.

TPC is also used for neighbour discovery, minimizing the energy consumption [35,36]. Unlike the work of Madan et al. [35], the one of Stabellini [36] considers the energy also in listening mode for the energy consumption calculation. Both approaches model the problem as MDP and they solve it offline via linear and dynamic programming. In the results, the authors analyse through numerical simulations the average energy consumption to discover the neighbours related to the density of nodes in the network. Madan’s results are compared against two schemas: an ideal schema of perfect knowledge and a simple one, where the power is doubled [35]. Instead, Stabellini’s results are compared against the optimal and suboptimal cases [36].

A centralized approach for energy savings is introduced in the literature, preventing the use of certain transmission power levels while a device is in a specific battery level range [41]. The problem is formulated as a discrete finite horizon MDP and is solved in a central node that collects all the needed information from the sensors. The central node calculates the optimal policy through the Bellman optimality equation. The battery lifetime of a sensor node is analysed and compared with other simpler policies, such as: not transmitting; transmitting with the highest available power; or transmitting with low and high power in the first and second half period, respectively.

Other solutions use MDP for harvesting energy protocols, where the transmission power is adjusted to get certain information [42,43,44].

RL has already been applied to WSNs for solving different aspects, as discussed in the survey of Kulkarni et al. [24]. Examples of these are the design and deployment of sensor nodes, routing and scheduling of sleeping time for energy saving techniques. In the survey, none of the contributions in RL have targeted QoS in WSNs, since energy efficiency is always prioritized. In addition, only one research direction uses RL for power control [33]. The work presents the actor/critic algorithm to maximize the ratio between throughput and energy consumption. The nodes learn the transmission power and modulation level to use based on the channel gain of the previous transmission and number of packets in the queue. Both the reward and the discounted return are iteratively averaged. The authors choose the Gibbs softmax as action decision strategy, which considers the probability of choosing an action knowing the state. The probability is also adjusted at every iteration. In the case of multi-node scenarios, the authors consider the interference in their model, which is an argument of the reward function. However, the nodes do not cooperate to reduce the interference. Furthermore, the interference is used as an input sequence of discrete values for numerical simulations. Similarly, also the other parameters, such as SNR, Signal to Interference plus Noise Ratio (*SINR*), buffer cost, distance between the nodes, are treated as a set of numbers to feed in the learning algorithm, instead of obtaining them as a result of stochastic models (e.g., localization, capture effect and fading) at every iteration. The network performance is compared with a simple policy, where the nodes transmit with the highest modulation possible, keeping a predefined SNR/*SINR* constant.

Another classification of RL considers the following schemes: MAC, cooperative communications, routing, rate control, sensing coverage and task scheduling [26]. Compared to the previous survey [24], MAC, rate control and task scheduling can be associated to the group of scheduling; routing and cooperative communications to the group of routing; and sensing coverage to the group of design and deployment. Two protocols apply RL to TPC [45,46]. In one work, the authors implement a collaborative routing protocol to deliver traffic flows from a source to a destination [45]. Whether the direct communication is not successful, relay communications are considered. The goal is to find the optimal routing path that provides certain performance, in terms of packet delivery ratio, delay and energy consumption. The nodes learn to take actions, such as transmitting with a variable transmission power or not transmitting. The strategy to choose the transmission power and the learning curve that brings the system to convergence are unfortunately not provided. In the other research track, RL is used for a completely different case, namely accessing the channel through either a short-range or a long-range radio transceiver, by selecting one of the four total transmit power levels [46]. The authors aim at reducing the energy consumption, which is the argument in the reward function when a packet is received, and packet loss, rewarding the system with a high negative value when a packet is lost. Both the methods use Q-learning as RL algorithm [45,46].

Few other solutions have used RL in WSNs for TPC. One of them is focused on delay sensitive applications for multi-hop communications [47]. The authors compare the network performance among the optimal policy, the centralized and the distributed systems. The centralized system considers the joint state and action of all the nodes involved, that are controlled at a central node. Instead, in the distributed system, the MDP is factorized and integrated in all the nodes. Each hop has its own tuple state–action–reward and value function that are updated at each epoch as defined in the work. The update at each node is calculated with the information of the value function from the other nodes. This information is transferred by the nodes themselves at a certain rate in packets with a variable length. Increasing the length and/or lowering the rate, the system is closer to obtain an optimal solution, but the overhead in the network increases as well, affecting the performance. Thus, the authors have considered a trade-off by considering an approximation of the exact value function, by reducing the transfer of information. The target of the system is to provide the delivery of the traffic flow under a certain delay constraint by defining the transmission power and the routing path. The MDP is solved through the actor and critic, RL algorithm. The performance shows that the distributed system performs better than the centralized.

The method on POMDP by Udenze et al. [40] is extended to RL [48]. Udenze et al. improve their previous work [40] by considering a more realistic environment in WSNs, which is initially unknown [48]. The main focus of the results is the comparison among the convergence time of three different solutions for an unknown environment, namely Monte Carlo, one step Temporal Difference (TD0) and Temporal Difference λ (TDλ), where the λ value averages the long term returns. Although the authors claim to study an unknown environment, they use a data set for the state transition probability. Similarly, the states of the environment correspond to the combination of transmission activity by the nodes in the network during a range of time slots. Hence, the number of states increases proportionally to the number of nodes and time slots. In addition, the number of nodes in the network must be known in advance to design the states. From a state, the agent can take three actions, specifically either transmitting at low or high power, or wait and transmit in the next time slot. The goal of the agent is to spend energy efficiently and avoid packet loss.

Le et al. [49] use Q-learning for topology control, applied to sensor nodes in order to keep the connectivity of the network with *k*-degree. The nodes exchange information regarding the transmission power used and communication range. Simulations in Matlab show energy, communication range and connectivity, comparing the method with spanning tree and fuzzy logic topology control, as well as with a network without topology control. The topology control with Q-learning is able to save energy, while keeping the desired grade of connectivity, with respect to the other schemes.

In addition, in the studies of Sung et al., Q-learning is used for ensuring the connectivity by adjusting the transmission power [50]. However, this work is specifically focused on improving the learning time of the Q-learning using a reward propagation method. In such a way, not only the Q-value of the observed state and executed action is updated, but also the Q-values of other state-action combinations are updated at the same time.

Q-learning applied to Wireless Body Area Networks (WBANs), to mitigate inter-network interference, increases the throughput and minimizes the energy consumption [51]. Kazemi et al. compare an approximated Q-learning, using radial basis functions, with two of their approaches that use fuzzy logic and game theory, respectively. The results, obtained through simulations, show that the RL approach outperforms the other techniques.

To summarize, different research tracks have studied the problem of TPC in WSNs through the formulation of MDP, but only few have solved MDP with online learning. Systems based on the offline approach are unrealistic and unfeasible for embedded systems because these are based on assumptions and statistical models. Indeed, the methods are evaluated either through theoretical work or numerical simulations. Most of the methods consider only one agent in one-to-one communications [34,42,46,51]. Instead, in the methods that study the network with multiple nodes, the system is designed in a fully joint state-action space. The coexistence of multiple links is taken into account only in the state and reward definition, considering either each node’s transmission mode combination (i.e., idle, transmitting with a power level) or the level of interference that is detected at the reception [33,40,41,48]. Such approach is also adopted for MDP and POMDP frameworks that are solved with online learning. This is implementable either in a centralized architecture, where all the components of the network transmit their local information to a central node, or by exchanging information among the nodes. However, in reality, the amount of data to be exchanged is unfeasible for dense wireless networks. The nodes deal with an unreliable environment. For this reason, we propose a TPC protocol that is defined as a Dec-POMDP solved with RL. Each node has its own local perception of the environment status, is independent, and does not require tight synchronization with the other nodes. Moreover, to the best of our knowledge, our protocol is the first one to consider cooperation in a multi-agent system for interference mitigation, providing connectivity in point-to-point communications, without exchange of information. The nodes cooperate indirectly to satisfy a common goal, equivalent to the same QoS requirements. Cooperation is possible thanks to a theoretical game approach, whose rules are taken into consideration in the reward calculations. In literature, Liang et al. use the collaboration for the routing protocol, focusing on the exchange of packets for the routing path decision [45]. Instead in the method of Lin et al., the nodes individually solve the MDP and exchange their local returns in the network, in order to decide the most efficient transmission power and routing path [47]. However, the work does not take into consideration simultaneous end-to-end communications, thus interference.

Unlike other related works, we test our protocol in a detailed, modular simulator and in real nodes. We analyse the convergence procedure in Q-learning by varying the factors of exploration and learning in time, as recommended in theory [38]. In all research literature using Q-learning, the parameters are set to a constant value. The results in each method are generally a comparison between optimal and suboptimal solutions (using the approximation method), or between the network performance using the specific method and constant transmission power. The problem formulation, the model of the environment, the goal of the system and the evaluated performance are different for every work. For this reason, the various methods presented in the literature have not been compared against each other.

## 4. Method

We adopt Q-learning for Transmission Power Control (QL-TPC), where the agent is the transmitter of a point-to-point communication and the environment is the wireless channel. The WSN is modelled as an MDP, single agent system, when there is only one transmitter in the network, and as Dec-POMPD, multi-agent system when there are more transmitters.

### 4.1. Q-Learning for Single-Agent Systems

MDP problems can be solved through 3 methodologies: Dynamic Programming (DP), Monte Carlo (MC) and Temporal-Difference (TD) [38]. We have selected the TD solution because it does not need a model of the environment (contrary to dynamic programming) and it is completely incremental, unlike Monte Carlo methods. TD methods provide returns online, at every step, while the value function is estimated using past learned estimates. They are distinguished in off-policy and on-policy. The difference resides in the dependency to a policy. In particular, although the off-policy methods are independent from the policy selected, the value function approximates to the optimum. Instead, on-policy methods are influenced by the policy chosen. We employ Q-learning, a well-known off-policy TD algorithm, which is integrated in a TPC protocol. The action-value function is renamed as Q-value, *Q*, and defined as follows:

(5) $$\begin{array}{c} Q_{k+1}\left(s_{k},a_{k}\right)=\left(1-\alpha_{k}\left(s_{k},a_{k}\right)\right)\cdot Q_{k}\left(s_{k},a_{k}\right)+\\ \alpha_{k}\left(s_{k},a_{k}\right)\cdot \left[r_{k+1}+\gamma \cdot \max_{a}\;Q_{k}\left(s_{k+1},a\right)\right], \end{array}$$

where $\alpha_{k}\in \left[0,1\right]$ is the learning factor at the *k*-th episode and γ∈[0,1] is the discount factor. α indicates the weight that the system gives to new rewards by updating the long-term Q-value. γ dims the contribution of the expected maximum Q-value in the new state. Q-learning is a lightweight algorithm that, in the single agent system, provides the convergence of the estimated action-value to the approximated optimal value with probability 1, considering the following assumptions [38]:

(6) $$\begin{array}{c} \sum_{k=1}^{\infty }\alpha_{k}=\infty \end{array}$$

(7) $$\begin{array}{c} \sum_{k=1}^{\infty }\alpha_{k}^{2}<\infty \end{array}$$

In addition to the assumptions in Equations (6) and (7), the system reaches convergence if all the actions are continuously explored [38].

### 4.2. Q-Learning for Multiple-Agent Systems

**Figure 2:**
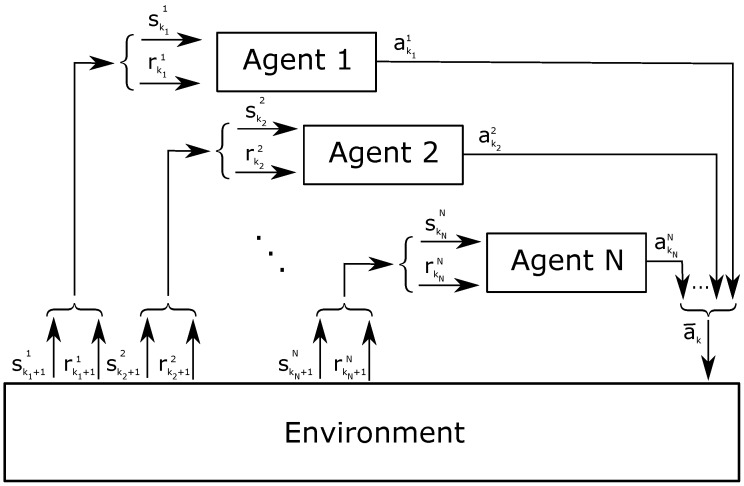
Multi-agent reinforcement learning schema.

When the system is composed by multiple agents, we adopt a Dec-POMDP model. Each agent is independent and interacts with a common environment, as depicted in Figure 2. Having independent learners, the complexity of the algorithm is proportional to the number of states and actions. Otherwise, learning in a fully joint state-action space, the agents need the knowledge of other agents’ decisions (i.e., actions or actions and rewards per transmitted packet) and keep track of action values for each one. If the shared information involves both actions and rewards, the complexity increases as $c∼n_{s}\cdot n_{a}^{n_{ag}}$, where $n_{s}$ is the number of states, $n_{a}$ the number of actions and $n_{ag}$ the number of agents [52].

The system follows the principles of game theory, such as the agents are players of a game (i.e., a mathematical model) that obtain a payoff by taking simultaneous actions [53]. The payoff is dependent to the target of the players. If they aim at improving only their own performance, the game is called competitive. Otherwise, if they gain a positive reward by helping each other, the game is cooperative. Moreover, if the players share the same target, they play a common interest game. In our model, the sensor nodes play a cooperative, common interest game, since the target includes transmission power reduction for interference mitigation and energy savings. At the same time, the nodes address the maximization of the PRR, over a window of *N* packets. At the end of a game, the system reaches an equilibrium, such as a combination of actions per player. The equilibrium is reached after an exploration phase, where the agents try different actions and get a reward from the environment. Therefore, the payoff of one agent is influenced by the decision of every component of the system. The optimal equilibrium is achievable if one agent knows exactly the actions and rewards of the other ones, while taking an action itself. This is not practicable in WSNs, due to an excessive increase in the complexity and memory requirements of the system, which would require continuous, instantaneous transfer of information via wireless communications. In Dec-POMPD, the grade of complexity to find the optimal solution is NEXP-complete, which means that the solution is provably not achievable [32].

Figure 2 illustrates the interaction of N agents with a common environment. Each agent *i* has a separate counter $k_{i}$ per episode, with 1≤i≤N, as well as its own 3-tuple parameters: state $s_{k_{i}}^{i}$, action $a_{k_{i}}^{i}$ and reward $r_{k_{i}}^{i}$. The action $a_{k_{i}}^{i}$ influences the environment in conjunction with the simultaneous actions $a_{k_{j}}^{j}$ of the other agents *j*, with 1≤j≤i−1 and i+1≤j≤N. The union of these actions forms the joint action $\bar{a_{k}}$. Among the actions $a_{k_{j}}^{j}$, one agent can be inactive and does not trigger its own cycle for Q-value updates. At the end of the episode $k_{i}$, the environment reveals the new state $s_{k_{i}+1}^{i}$ and reward $r_{k_{i}+1}^{i}$ to the agent *i*.

### 4.3. Q-Learning Transmission Power Control (QL-TPC)

In this section, we describe the proposed protocol QL-TPC, where the Q-learning algorithm is integrated with TPC.

#### 4.3.1. MDP Parameters Definition

In our system, the episodes are the transmission of *N* packets in a window *W*. Therefore, the state–action–state transition and reward acquirement happen after each *k*-th window. During one window, the same action is taken. The actions are chosen following the ϵ-greedy strategy, based on the exploration factor ϵ∈[0,1]. If ϵ≫0, the actions are taken randomly ($a\in U(a_{min},a_{max})$) with high probability, in order to learn how the environment reacts to different available decisions. Otherwise, when ϵ≪1, the system exploits the knowledge acquired by selecting the actions that have given the maximum action-values. More specifically, the actions are chosen by comparing a random value x∈U[0,1] with the parameter ϵ, which can vary in time:

$$a=\left\{\begin{array}{cc} U(a_{min},a_{max}) & \mathrm{if}\;x\leq \epsilon \\ \arg\;\max_{a}\;Q_{k}\left(s_{k},a\right) & \mathrm{if}\;x>\epsilon \end{array}\right.$$

The actions represent the available transmission power levels: $a_{k}=p_{tx,k}$. The state is a combination of rounded averaged retransmissions and Clear Channel Assessments (CCA) attempts, over *W*. The state describes the status of a link, providing an evaluation of interference at both the transmitter (i.e., CCA) and receiver sides (i.e., retransmissions). The state is expressed as:

(8) $$\begin{array}{c} s_{k}=∥\bar{retr_{k}}∥+∥\bar{cca_{k}}∥\cdot \left(n_{retr}+1\right), \end{array}$$

where $n_{retr}$ is the maximum number of retransmissions, $\bar{retr_{k}}$ and $\bar{cca_{k}}$ are the number of retransmissions and CCA attempts, at the *k*-th episode, respectively. Each retransmission is attempted for a number of maximum CCA, $n_{CCA}$, which is reset after the packet is sent. Therefore, the total maximum CCA for one packet transmission is $n_{CCA}^{tot}=n_{CCA}\cdot \left(n_{retr}+1\right)$. The rewards are defined as a combination of PRR, quantized linearly over a $m_{prr}$-level scale, and $n_{ptx}$ power levels; higher reward suggests higher PRR and lower power level, whereas lower reward suggests lower PRR and higher power level. The calculation of the reward is given by:

(9) $$\begin{array}{c} r_{k}=\Delta \cdot \left[\left(prr_{k}^{q}-1\right)\cdot n_{ptx}+\left(n_{ptx}-p_{tx,k}\right)-\frac{m_{r}}{2}\right], \end{array}$$

where Δ is the quantization step size factor between two consecutive quantization levels. $prr_{k}^{q}$ and $p_{tx,k}$ are the quantized PRR and power levels at the *k*-th window of transmitted packets. $m_{r}$ is the number of quantization levels of the reward and is expressed as $m_{r}=m_{prr}\cdot n_{ptx}$, with $m_{prr}$ the number of quantized PRR levels.

The nodes generate a Q-matrix that contains the Q-values of state-action combinations, where the states and actions are the rows and columns of the matrix, respectively. The Q-values are initialized with null values. The transmission power is initialized with the minimum value.

#### 4.3.2. Software Architecture and Flow Chart

**Figure 3:**
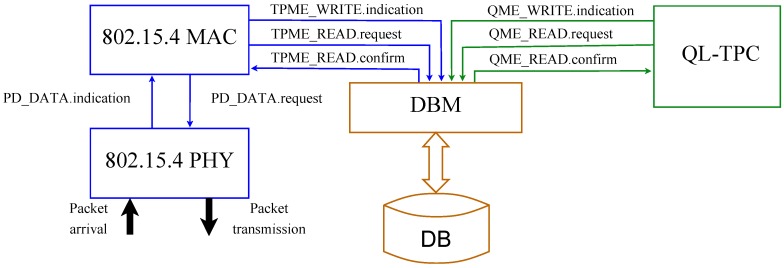
Software architecture.

**Figure 4:**
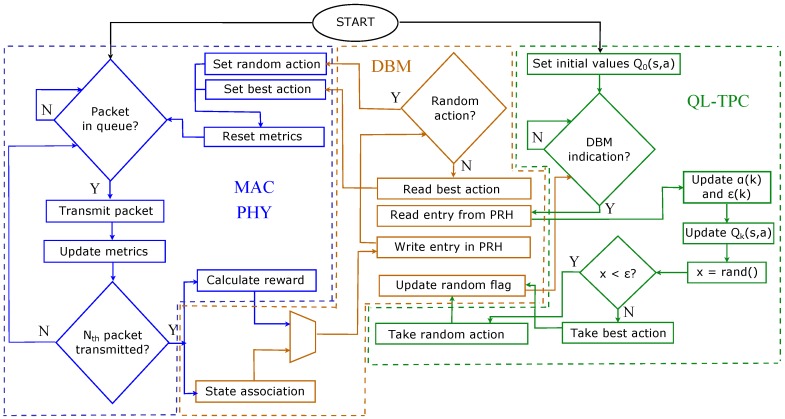
Flowchart.

The TPC protocol, depicted in the software architecture in Figure 3 and flow chart in Figure 4, is implemented in 3 blocks: the IEEE 802.15.4 PHY and MAC layers, Database Manager (DBM) and QL-TPC. The former and the latter communicate with the DBM for gathering, saving and sharing data. The MAC layer from the standard is customized to interact with the two new modules, DBM and QL-TPC. Looking at Figure 3, the 802.15.4 MAC module indicates to DBM the data to convert into the state and reward for the algorithm through the Transmission Power Management Entity (TPME), using the TPME_WRITE.indication message. However, it requires from DBM the transmit power to set for the next transmission via TPME_READ.request. DBM provides the power level in the TPME_READ.confirm message, that is then forwarded to the 802.15.4 PHY module by a PD_DATA.request message.

The Q-learning algorithm and ϵ-greedy strategy are implemented in QL-TPC, therefore the updated Q-values and the action strategy to pursue (i.e., random or maximum) are indicated through the Q-value Management Entity (QME), via the QME_WRITE.indication message to DBM. QL-TPC requests from DBM new state–action–reward triplet, when available, in order to update the Q-values, by sending QME_READ.request. Then, the reply is contained in a QME_READ.confirm message. DBM handles a database (DB) that saves the information obtained by the MAC and QL-TPC modules (i.e., an array of values containing the old and new Q-states, action taken and reward obtained; the Q-matrix; and the flag to indicate the action strategy) [54].

The IEEE 802.15.4 MAC and PHY models and QL-TPC are two blocks of processes that run independently. Their cycle of operations are detailed in the flowchart of Figure 4. The MAC and PHY block starts when the transmitter has a packet in the queue ready for sending. In the case of the first packet to be sent, a random action is selected and the state at the initial iteration is the lowest ($s_{0}=0$). When a node has buffered a packet for transmission, it accesses the medium through Carrier Sense Multiple Access with Collision Avoidance (CSMA/CA) up to a standard maximum $n_{CCA}$. If the CCA is successful, the packet is transmitted and an acknowledgement (ACK) is expected. If the ACK is not received, a copy of the packet is retransmitted, repeating for a maximum of $n_{retr}$ times. If the $n_{CCA}$ or $n_{retr}$ are exceeded, the packet is dropped. The same transmission power is used for *N* consecutive packets, whereas the CCA and retransmissions are recursively averaged and rounded to the closest integer. After the transmission of the *N*-th packet, the reward $r_{k+1}$ is calculated, as well as the CCA and retransmission values are associated to a state $s_{k+1}$ in DBM. $s_{k+1}$, $r_{k+1}$ and $s_{k}$ are inserted in one entry of the Packet Reception History (PRH) table in DBM, which is required by QL-TPC to update the Q-values. At this point, DBM checks a flag that indicates whether to select a random action or to exploit the action with the maximum Q-value. Such action is used for the transmission of the following *N* packets, setting the same power. The MAC layer communicates the chosen power level to the PHY level that is set for future transmissions. The QL-TPC process starts with the initialization of the $Q_{0}\left(s,a\right)$ values to 0. Afterwards, the process waits for indications by DBM whether the PRH table is not empty. In that case, QL-TPC reads the values in the first entry of the table, updates the variables α(k) and ϵ(k) to referred values, and calculates the new value for $Q_{k}\left(s,a\right)$. Subsequently, the ϵ-greedy strategy is used to decide the action for the next round of transmissions. Both processes are repeated for the entire simulation time.

## 5. Simulations

### 5.1. Setup

**Figure 5:**
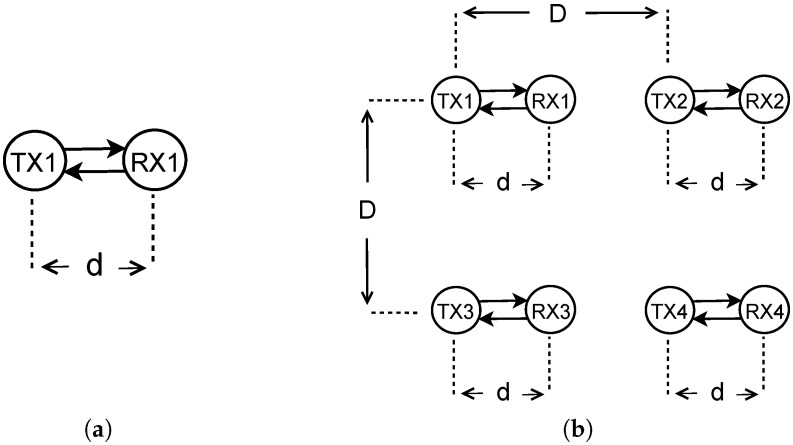
WSNs with Manhattan grid topology: (**a**) single agent—one wireless communication without interference; and (**b**) multi-agent—four interfering pairs.

Our TPC protocol is implemented in the Network Simulator NS3, a modification of release ns3.23 (https://www.nsnam.org/ns-3-23/), considering different scenarios. We set up a WSN with a variable number of cells structured in a Manhattan grid topology (Figure 5). A cell of the grid contains two nodes, distributed horizontally, where the transmitter is the left node at a distance *d* from the receiver. The distance between the cells, *D*, is referred to as the distance between the transmitters of the node couples. The transmitter generates a stream of packets with Poisson distribution and variable inter-arrival time μ. The size of the packets’ payload is constant and equal to 50 bytes, over the maximum payload of 123 bytes allowed in the standard 802.15.4 [55]. A summary of all the parameters involved in the setup are listed in Table 1 with their related values.

The devices are modelled as nodes of a low range wireless personal area network, using the 802.15.4 standard for PHY and MAC communication layers, and the 2.4 GHz Industrial, Scientific and Medical (ISM) unlicensed bandwidth over 16 channels of 2 MHz each. Channel 26 is used in this work. The interconnection architecture is modelled up to the MAC layer, so the communications are point-to-point. The MAC layer is modified in order to interact with QL-TPC and DBM. The environment is modelled with the site-general of ITU-R P.1238-7 for propagation loss in a building [56], and Nakagami for fast fading model [57]. The packets at reception are either accepted or discarded, depending on the implemented error model. In the latter, the Packet Error Rate (*PER*) is provided through the calculation of the Bit Error Rate (*BER*) as per the 802.15.4 standard [55]. The *BER* is dependent to the *SINR*:

(10) BER=815·116·∑k=216−1k16ke20·SINR·(1k−1)

(11) $$\begin{array}{c} PER=1-{\left(1-BER\right)}^{n_{bits}}, \end{array}$$

where $n_{bits}$ is the number of bits received. A packet is dropped if the *PER* is lower than a random value uniformly distributed between 0 and 1. The *SINR* is calculated as the ratio of the received signal power and the sum of noise and interference. Both the elements are known to the physical model. The former is calculated based on the transmission power, the attenuation and the fading model, applied to the signal. Similarly, the interference is calculated as the sum of the interfering signals’ power at the reception. Each interfering signal belongs to separate links, where the fading factor is random, following the Nakagami distribution, and the attenuation factor depends on the distance between the receiver and the interferer. Instead the noise is the Additive White Gaussian Noise (AWGN), whose power is equal to $k_{B}\cdot 290$, as per the thermal noise.

**Figure 6:**
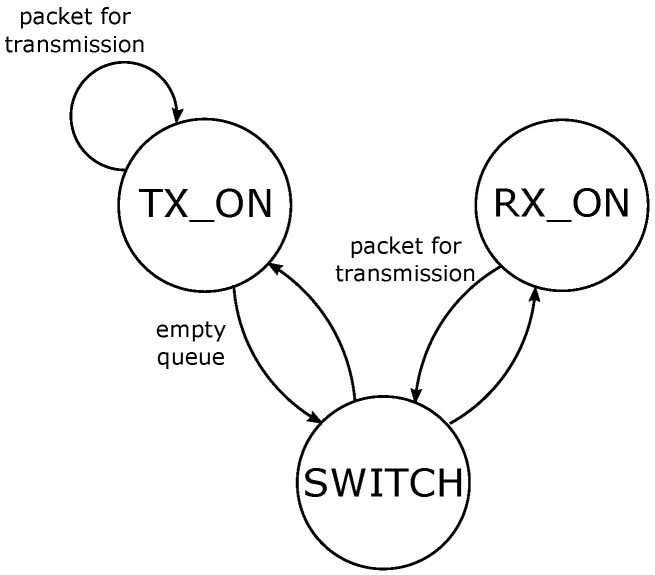
Finite state machine of the energy model.

The physical layer is responsible for the management of the radio activity. It decides whether to switch on the radio for either transmission or reception, or to switch off the radio and keep it idle. Depending on the status of the radio, the device supplies different currents, and thus consumes dissimilar energies. The energy is calculated as E=V·i(rs)·T, where i(rs) is the current used during a period *T* in a certain radio status rs, and V is the supply voltage. The possible statuses are: TX_ON and RX_ON, if either the transmitter or the receiver are turned on; SWITCH, as a transient mode, when the radio is switching between TX_ON and RX_ON. The radio is constantly in the receiving mode, RX_ON, and switches in the transmitting mode, TX_ON, only when there are pending messages to be sent. The finite state machine of the energy model is shown in Figure 6. The specifications for the supply voltage and current at each radio status are referred to the AT86RF233 low power transceiver [58] and listed in Table 2. The current in the transmission mode, $i_{tx}\left(p_{tx}\right)$, follows a linear current model taking the transmission power as an argument:

(12) $$\begin{array}{c} i_{tx}\left(p_{tx}\right)=\frac{p_{tx}}{V\cdot \eta }, \end{array}$$

where η is the efficiency of the amplifier that is adapted to obtain the same referred value for the power equal to 0 dBm, as in [58]. Therefore, η is equal to 2.8%. The supply voltage is equal to 3 V.

The WSN is analysed in two different cases: HG and QL-TPC. In both cases, the transmission power for the ACKs is selected at random, uniformly distributed among the available power levels, at the beginning of one simulation and kept constant. In the HG case, all nodes use the same amount of transmission power, among the 20 available levels. The lowest and the highest transmission powers are equal to −35 dBm and 10 dBm. For each power level, the performance parameters are averaged by R=10 runs of one simulation. Each run uses different sub-stream of pseudo-random numbers, within the whole stream generated by one seed in NS3. The random variables are: power level selection for ACKs, packet generation time, fading per link and packet drop. In the QL-TPC case, all the nodes run the Q-learning algorithm. Each node obtains one value per parameter related to one power level. Such power level is obtained for a specific node, during the testing phase, by a weighted average of the transmission power used for all the *R* simulation runs. Therefore, the average transmission power in dBm by one node is calculated as follows:

(13) $$\begin{array}{c} \bar{P_{tx}}=\frac{\sum_{r=1}^{R}\sum_{l=1}^{n_{ptx}}p_{tx,l}^{r}\cdot n_{l}^{r}}{\sum_{r=1}^{R}\sum_{l=1}^{n_{ptx}}n_{l}^{r}}, \end{array}$$

where $p_{tx,l}^{r}$ is the transmission power in dBm, corresponding to the *l*-th power level and *r*-th simulation repetition, and $n_{l}^{r}$ is the number of occurrences of $p_{tx,l}^{r}$ usage. In this case, there are two more random variables to consider: ϵ-greedy strategy and power level in the exploration phase. The performance is analysed through three parameters: PRR, latency and energy per bit. PRR is a ratio between the acknowledged and transmitted packets over a window of N=10 packets; the latency is the time difference between the generation and the acknowledgement of a packet; the last term is the energy consumed per transmitted bits.

The simulations are divided into seven periods of 600 s and one of 1800 s, as shown in Table 3. The simulation time is divided in three phases: learning, convergence and testing. In the learning phase, we split the time into four periods of 600 s each. The learning factor is kept high, so that the system learns rapidly the impact of the actions on the environment. The system switches from a purely exploratory approach to the exploitative one, with a low margin of exploration. The drawback is that the Q-values may oscillate over a broad range of values. Hence, in the convergence phase, over three equal periods of 600 s, the system keeps learning at a decreasing slow rate, while exploiting the learnt data. In this way, the agents slowly converge to the Q-value of one action, reducing the variance of the oscillations. Finally, in the remaining 1800 s, the system is tested and evaluated. At this point, the nodes have their final Q-matrix, where for each Q-state, the transmission power level with the maximum Q-value is associated. The combination of ϵ and α for the different periods and their duration is chosen based on our study, where the effect of different values has been evaluated on the convergence of the Q-value. We select the combination that brings the system to converge with small variance of the selected actions per simulation run, following the aforementioned procedure.

### 5.2. Results

QL-TPC is studied first in a scenario without interference, where there is only one transmitter and one receiver, in the network of Figure 5a, and then in a cross-interfered network among eight nodes (four transmitters and four receivers), as per Figure 5b. The former scenario is the equivalent of a system with one single agent in a Nakagami fading environment. Instead, the latter is a multi-agent system in a more complex environment, influenced by the interference of different agents. The results in QL-TPC are compared with HG, where the transmission power is constant for each node.

#### 5.2.1. Single Agent WSN

**Figure 7:**
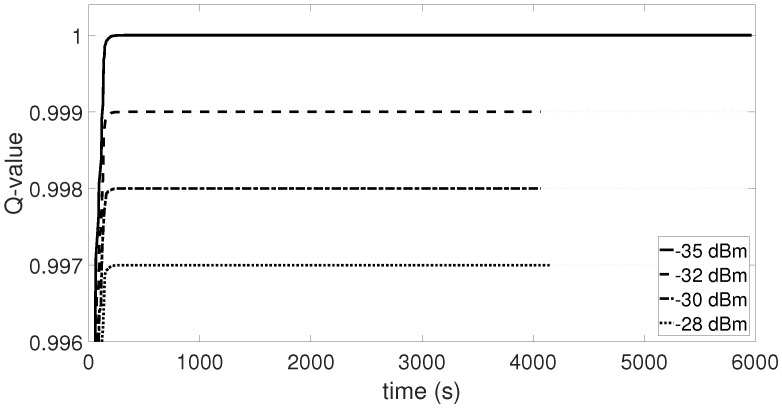
Normalized Q-value of the single agent in a network of two nodes (2 m node distance) and average inter-arrival time of 25 ms, where the trend is focused on the lowest transmission power levels.

The agent is found only in Q-state equal to zero that expresses the condition with null retransmissions and CCA reattempts. During this state, Figure 7 shows the normalized Q-value convergence of the transmitter at 2 m distance from the receiver for the four lowest transmission power levels, using an inter-arrival time of 25 ms. The separation among the power levels is fixed and represents the upper-bounds for each level. Such separation is the outcome of the reward function, linearly decreasing as the power level increases, as per Equation (9). The prevailing transmit power is the lowest, equal to −35 dBm.

**Figure 8:**
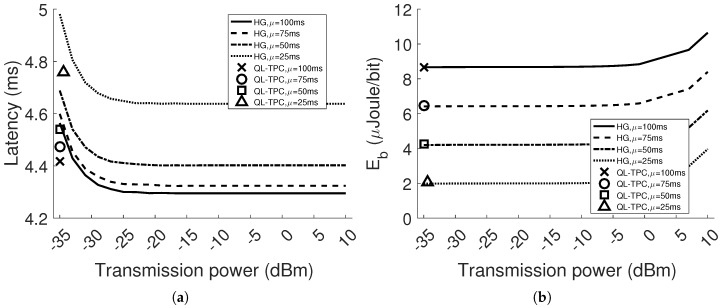
Performance in WSN with 2 nodes during the testing period (2 m node distance): average latency (**a**); and energy consumption per bit (**b**).

**Figure 9:**
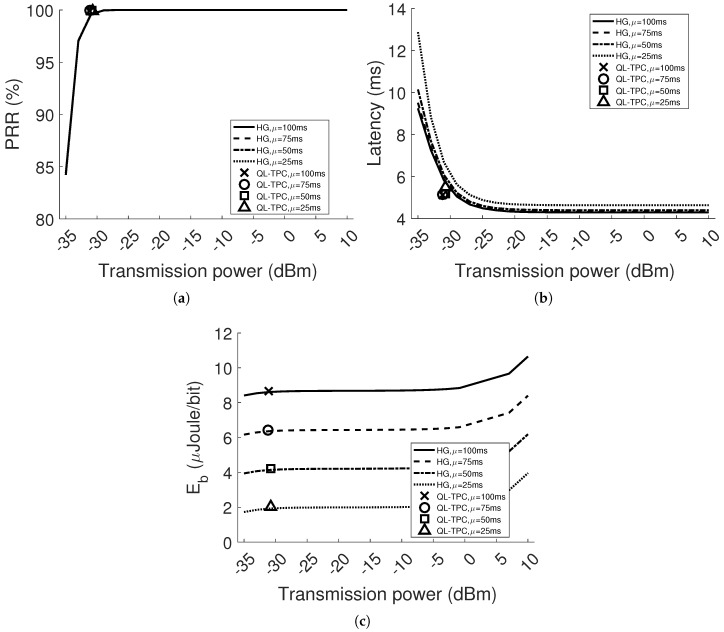
Performance in WSN with two nodes during the testing period (4 m node distance): average PRR (**a**); average latency (**b**); and energy consumption per bit (**c**).

In Figure 8 and Figure 9, we analyse the PRR, latency and energy consumption per bit of the WSN in two different conditions, where *d* is equal to 2 m and 4 m, respectively, changing the average inter-arrival time value, μ, of the Poisson distribution for the traffic generation. Each curve of the homogeneous case shows the global performance, as the average among all the nodes. The results of the QL-TPC case are plotted with marks, where one mark represents the value of the transmitter’s performance parameter, corresponding to the average transmission power used as per Equation (13). The simulations run for 500 s in the case of HG and 6000 s in the other case of QL-TPC, as stated in Table 3. When *d* is set to 2, the average PRR is 100% in HG, independently of μ and transmission power used, since there is no interference and the receiver is in the communication range of the transmitter, regardless of power. In this simplest case, the QL-TPC is also able to obtain 100% of PRR, choosing the lowest average transmission power equal to −35 dBm. When the average inter-arrival time is set to 25 ms, the selected transmission power is slightly higher at −34.40 dBm. Although the average PRR is constant for all the transmission power levels, the average latency in Figure 8a decreases when the transmission power changes from −35 dBm to −25 dBm with constant value of μ. Moreover, the average latency raises at a fixed transmission power level and at increasing inter-arrival times. This is due to the higher average waiting time of the packets in the queue. The latency obtained by the agent using QL-TPC is better than in the homogeneous case for the selected transmission power by a factor that varies between 0.1 and 0.2 ms. Nevertheless, it is higher than the minimum latency of the homogeneous network. For the purpose of our protocol, it is not relevant because the focus is only on the maximization of PRR. The third parameter that we analyse is the energy per bit (Figure 8b). The node consumes less energy if the transmitter uses the lowest power level. Indeed, the current used by a device is proportional to the transmission power, as per Equation (12). The values of energy per bit in QL-TPC at the selected transmission power levels correspond to the minimum values in the homogeneous case. Therefore, the device learns to save energy using QL-TPC.

When the distance *d* doubles, the performance in HG worsens at the lowest transmission power levels. The attenuation is higher and more bit errors occur. Indeed, PRR varies from 84% to 100%, increasing the transmission power from −35 dBm to −27 dBm, respectively. In the same range of power, the average latency is higher and reaches the plateau at −15 dBm for the minimum values, instead of −23 dBm when d=2 m. The energy per bit is lower for values of transmission power between −35 dBm and −17 dBm. In such a situation (d=4 m), the conditions are changed and we test the protocol as before. The node learns to use an average transmission power of around −31 dBm with a standard deviation between 0.04 and 0.2 among different values of μ. The environment is found in states 0 and 5. Both PRR and latency are improved with respect to the homogeneous network at the same power, as depicted in Figure 9a,b. The PRR for all the inter-arrival time values in QL-TPC is around 99.9% compared to values around 99.6% in HG. The latency is decreased by 1 ms to 5.5±0.2 ms, when μ is equal to 25 ms, and by 0.7 ms to 5.2±0.3 ms, when μ is equal to 100 ms. The energy per bit values intercept the curve of the homogeneous case. The values are 8.65, 6.44, 4.23 and 2.03 μJ/bit, using the inter-arrival time from 100 ms to 25 ms, respectively, where the standard deviation is in the order of $10^{-5}$.

#### 5.2.2. Multi-Agent WSN

**Figure 10:**
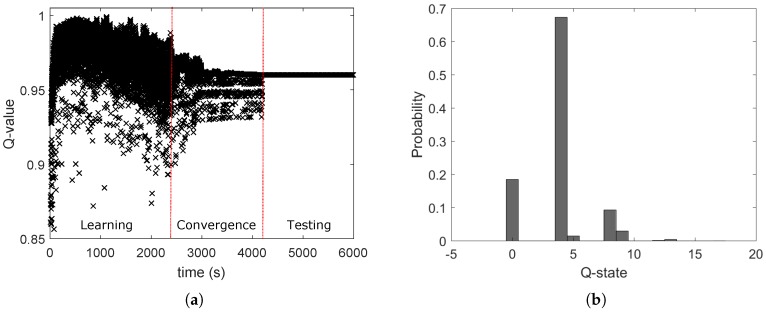
Q-value of TX1 in the multi-agent system, while residing in the Q-state equal to 4 (**a**). The probability of residing in all Q-states is shown in (**b**).

The multi-agent WSN is composed of four node pairs distributed in a Manhattan grid topology (Figure 5b). The transmitters of each couple represent the agents of the QL-TPC protocol. Each agent observes a dynamic environment that is furthermore modified by the action of other agents. One action is a transmission that enables communication between two nodes but also creates interference with other nodes. In Figure 10, the outcome of the Q-learning process is illustrated. The distance *d* for each couple is equal to 2 m. Figure 10b presents the probability that the environment is in a certain state during a simulation, when the inter-arrival time μ is equal to 25 ms. The most likely state is Q-state 4 that is further evaluated in Figure 10a for TX1, as illustrated in Figure 5b. The Q-value is showed, marking the separation of the trend among the phases of learning, convergence and testing, as detailed in Table 3. During the learning phase, the Q-value is in a transient period that starts with a rise and then continues with an oscillation. The agent explores the effect of every action on the environment using a high learning factor. Therefore, the choice of each action provides different variance of the oscillations. The oscillations are reduced in the next phase of convergence, since the learning factor is lowered. In addition, the exploitation of the best action is more frequent than the explorations. Indeed, the predominant contribution is marked and the wider oscillations are due to the small margin of exploration. In this phase, the curve converges to the maximum Q-value obtained by one action. The selected transmission power is equal to −32 dBm and is then used during the whole period of the testing phase. In the latter phase, the network performance is evaluated. Similarly, other transmission power levels are selected for other states, as shown in Figure 10b.

**Figure 11:**
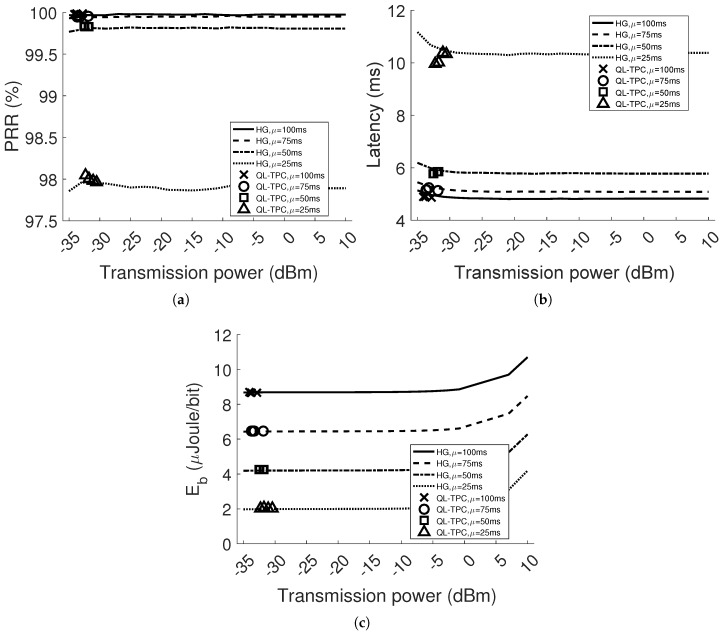
Performance in WSN with eight nodes during the testing period (2 m node distance): average PRR (**a**); average latency (**b**); and energy consumption per bit (**c**).

**Figure 12:**
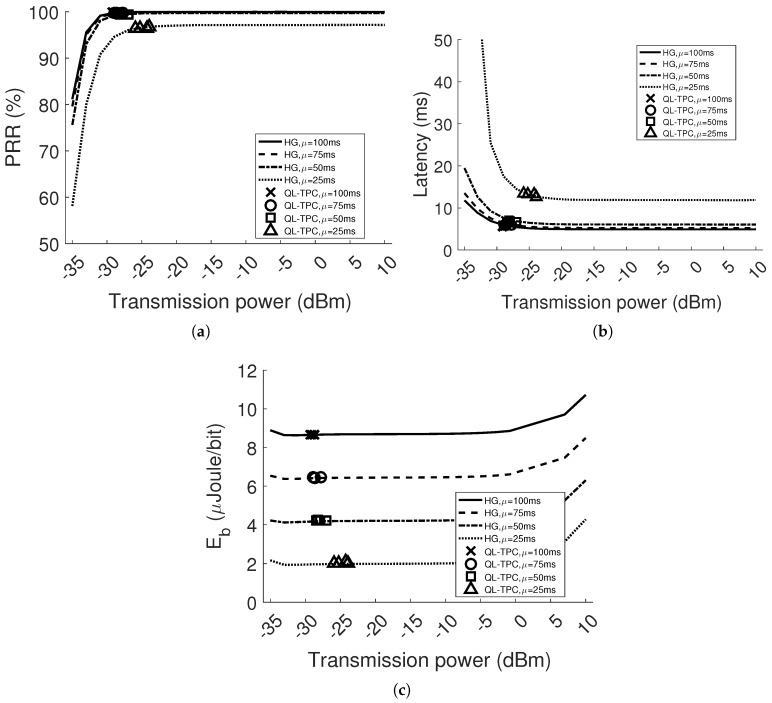
Performance in WSN with eight nodes during the testing period (4 m node distance): average PRR (**a**); average latency (**b**); and energy consumption per bit (**c**).

Figure 11 and Figure 12 depict the results when *d* is equal to 2 m and 4 m, respectively. As expected, the performance generally deteriorates compared to the single agent system. This happens especially when μ is equal to 25 ms. Indeed, considering the homogeneous case in Figure 11a, the PRR for every power level drops to around 98%. However, in Figure 11b, the latency is higher, starting with 11.2 ms using the lowest transmission power level and stabilizing to around 10.3 ms. Raising the distance between the nodes, the performance worsens, since the signal strength in SINR decreases and the bit error rate increases. Figure 12 reveals the decay of the performance in the homogeneous network at the lowest power levels. The combination of the attenuation and interference brings the PRR to drop and the latency to boost, while using low transmission power, between −35 and −25 dBm. For instance, the PRR drops by 31% and 40% in the case of the transmission power set to −35 dBm and the lowest inter-arrival time, comparing Figure 9a and Figure 11a, respectively, with Figure 12a. For the same case, in Figure 12b, the latency has a pick of 16 s. This is due to the higher packet generation rate with respect to the packet transmission rate, such as the average retransmissions and CCA attempts are increased to 1.5 packets and the average queue occupancy to 631 packets. The aforementioned scenarios present different environmental conditions than in the previous section. Figure 11 and Figure 12 show how QL-TPC performs by adapting the agents’ decision to different environment. The marks per inter-arrival time are four and correspond to the average value of each transmitter per performance parameter, at the selected average transmission power. The values of PRR, latency and energy per bit obtained with QL-TPC are similar to the homogeneous network ones per selected transmission power. Increasing the distance *d* to 4 m, QL-TPC achieves transmission powers shifted towards higher levels, preventing low SINR. In Figure 12a, when μ=25 ms, PRR is around 96.5%, higher than the requirement of 95%, between power levels −25.9 and −23.9 dBm. Such PRR value is similar to the value of HG at the same power level and lower than the maximum 97.17% at 7 dBm. In Figure 12b, the average latency among the transmitters is 13.13 ms compared to the minimum latency in HG that is equal to 11.83 ms at 7 dBm. The energy per bit is 2.03 μJoule/bit against the minimum 1.93 at −33 dBm and the maximum 4.28 at 10 dBm. In general, the results show that using QL-TPC, the PRR is always within the constraint range 95–100%. Comparing our protocol with the optimal PRR (maximum) and latency (minimum) in HG, at the same inter-arrival time, the PRR values are 0.1–0.7% lower and the latency is higher, up to the 14%. However, the optimal network performance is obtained at higher transmission power levels than the ones selected by QL-TPC, the energy per bit is reduced in QL-TPC by 19.22–52.57% of the maximum value in HG at high power values. At the same time, the energy per bit in QL-TPC is higher than the minimum value in the homogeneous case by 0.35–5.18%, but achieved at lower transmission power levels, where PRR and latency deteriorate. Our protocol achieves a trade-off between network performance and energy consumption.

**Figure 13:**
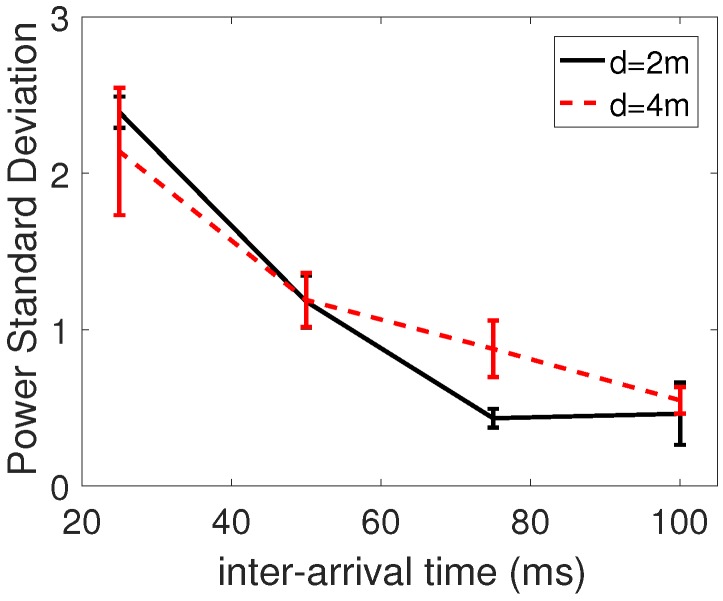
Power standard deviation in a WSN with eight nodes.

The transmission power selected by QL-TPC is calculated as per Equation (13), an average that considers each contribution per Q-state and simulation re-run. The standard deviation of the transmission power is shown in Figure 13 for cases of *d* equal to 2 m and 4 m. It is averaged among the values of the 4 transmitters and its standard deviation is delimited by interval bars. The highest values are in the intervals 2.4±0.1 (d=2 m) and 2.1±0.4 (d=4 m), in the harshest conditions, when the interference is maximum. Otherwise, in the other conditions, the standard deviation is less than 1.4 dBm.

#### 5.2.3. Energy Consumption Analysis

The energy consumption is directly related to the power and time used by a device while performing specific tasks. Our model excludes sleeping techniques, thus, when the queue for packets is empty, the receiver is kept on. Indeed, the energy is mostly consumed in reception mode, and overall varies based on the power intensity and duration of the transmissions.

**Figure 14:**
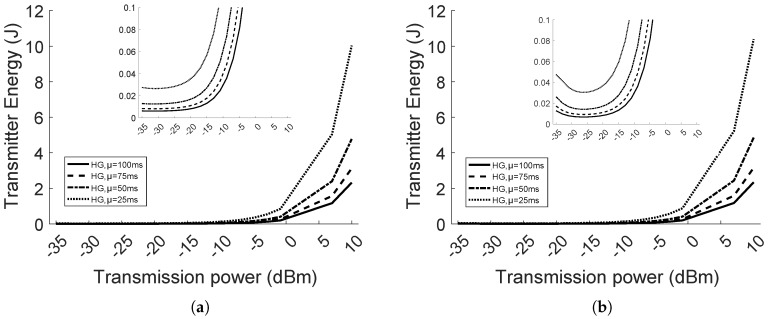
Transmit energy consumption, zooming on the lower values, of the homogeneous network with eight nodes and *d* equal to: 2 m (**a**); and 4 m (**b**).

**Figure 15:**
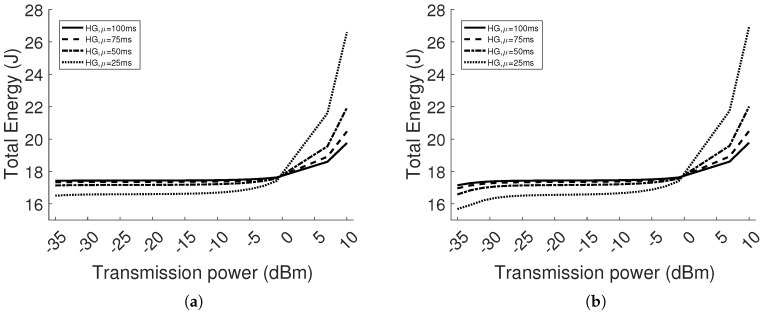
Total energy consumption of the homogeneous network with eight nodes and *d* equal to: 2 m (**a**); and 4 m (**b**).

The transmission duration is influenced by the rate of packet generation and retransmissions as a consequence of attenuation, fading and interference. In this section, we provide the details of the average energy consumption in the homogeneous network with eight nodes. Figure 14 shows the energy consumption only in the transmission mode, while Figure 15 illustrates the total energy consumption in all the modes (i.e., transmission, switch and reception). Looking at Figure 14a, where d=2, the energy consumed by the nodes in the transmission mode has a monotone increasing trend. Instead, in Figure 14b, where d=4, the energy consumption has a convex shape. In the latter case, the energy in transmission mode, using the power between −35 dBm and −27 dBm, is higher than in the case where d=2, because more retransmissions are needed to successfully deliver the packets, given the increased attenuation in the link (i.e., higher *BER*). However, in the same range of transmission power levels, the total energy is lower, as per Figure 15a,b. It is explained by the fact that spending more time in transmission mode, in a constant period of observation, is beneficial for the total energy consumption, when the transmit power is lower than 0 dBm. In the latter case, the current draw is higher at reception than transmission. Moreover, the node is at reception mode by default, if the buffer of packets to transmit is empty. For the same reason, in Figure 15, the total energy consumption, up to 0 dBm, has the lowest trend when the inter-arrival rate μ=25 ms, and increases as μ raises. The trend is reversed after 0 dBm.

## 6. Experiments

In this section, we provide the experimental setup and results of QL-TPC for a point-to-point communication. The system is the same as in Section 4.1, but the setup is different than the one used for the simulations. We use TelosB sensor nodes with Contiki OS. TelosB has 48 kB of program flash, 10 kB of data RAM and 1 MB of external flash. The nodes are connected to a cabled test-bed, which is formed by BeagleBone Black (BBB) devices linked via Ethernet to a server, through a Virtual Private Network (VPN). Each node is connected via USB to one BBB. Using the test-bed, we are able to store and analyse reliably the traffic that is generated, transmitted, received and lost through the ether. The TelosB nodes run the Contiki program that implements the QL-TPC protocol. In Contiki, we use the unicast routing protocol in the network layer, and Contiki-MAC in the MAC layer. The latter is an asynchronous radio duty cycling protocol, which has been created following the characteristic of the IEEE 802.15.4 standard. In Contiki-MAC, the radio is in sleeping mode by default (i.e., the radio is off) and it turns on every 0.5 ms for 0.192 ms. The transmitter sends copies of the same packet every 0.4 ms. Using this procedure, the nodes save energy and, at the same time, are able to communicate [59]. The program allocates memory in the device as follows: around 28 kB for the executable code, 118 bytes for the initialized data and 6.8 kB reserved for uninitialized data. The former is written in the ROM, the second in the EEPROM and the latter in the RAM. The BBBs use a Python program that reads the data from the TelosB and forwards it to a central server via a RabbitMQ messaging system. The format of the data from the TelosB to the server is JSON. The server runs a Python application that parses the JSON data and makes a CSV file, which is used for data analysis in MATLAB. The experiments are performed in the lab. The nodes are distant 8 m between each other. The choice of ϵ in time follows an exponential function $\epsilon =\lambda^{N}$ with λ=0.998. The value for α and γ is constant and set to 0.8. The number of actions are 8, which correspond to the transmit power levels of the TelosB. The state is determined based on the number of retransmissions, number of CCA attempts and the latency as follows:

(14) $$\begin{array}{c} s_{k}=L^{2}\cdot ∥\bar{cca_{k}^{q}}∥+L\cdot ∥\bar{retr_{k}^{q}}∥+\bar{lat_{k}^{q}}, \end{array}$$

with L=4 the number of quantization levels, $\bar{cca_{k}^{q}}$, $\bar{retr_{k}^{q}}$ and $\bar{lat_{k}^{q}}$ the average quantized number of CCA attempts, retransmissions and latency of the *k*-th episode, respectively. The quantization association is shown in Table 4. The latency is classified in low, medium and high, based on the values obtained during the experiments. They are associated to the range equal to the cumulative difference between the maximum and the minimum, divided by 3. When a packet is lost, the latency is NA and is ignored for the average in the window *W*.

Finally, the reward is calculated as the PRR over *W*, in its continuous form. The experiment is 750 min long. The inter-arrival time of the packet generation is equal to 3 s. The power level used to send ACKs is constant and set to 7. The size of the Q-matrix is equal to 2.048 kB, given that the matrix contains an amount of float numbers equivalent to the multiplication of $n_{s}\cdot n_{ptx}$. The values of the parameters involved in the experiments are listed in Table 5.

**Figure 16:**
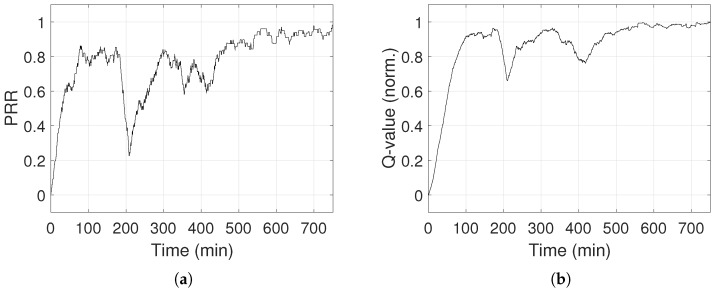
PRR (**a**); and Q-value (**b**) obtained through experiments in the lab.

In Figure 16, PRR and Q-values rise over time, showing that the system learns and improves its performance. When the system is mostly exploring (i.e., ϵ≫0), PRR is unstable, but as ϵ drops, PRR stabilizes between 90% and 98%. PRR never reaches 100%, because the system keeps exploring, since ϵ never reaches 0. Exploring gives the possibility to choose power levels that result in low PRR and low Q-values. When ϵ drops below 0.1 (i.e., after 550 min), PRR remains above 87%. As a last note, PRR and Q-value drop in Figure 16 after 200 min, which is explained by the fact that someone entered in the lab. The environment changed and its representation in the system moved into a new state that was not explored previously, causing the drop and a new learning curve. Differently from Section 5, the convergence is longer because the inter-arrival time of packet generation is 3 s, thus the PRR is calculated around every 30 s. We also analyse the energy consumption of the transmitter node and compared it to the homogeneous case, when the maximum transmission power is set. The values of the parameters for the energy calculation are taken from the datasheet of the transceiver CC2420 [60]. The nodes can be in four different operational modes: sleep, idle, transmission and reception. In the sleep mode, the power supply is turned off. The idle mode in TelosB is equivalent to the power down mode in CC2420 where the crystal oscillator, FIFO buffer and RAM access are disabled. During the transmission and reception mode, the node transmits and receives, respectively. The nodes are in sleep mode for 61.6% of the time. The transmitter sends the data every 3 s, with a payload of 8 bytes added to 6 bytes of header. The time in the transmitting mode, $t_{tx}$, is calculated by the number of packets that are transmitted and retransmitted during the entire time of the experiments (i.e., 750 min), at the data rate of 250 kbit/s. Similarly, the time in reception mode, $t_{rx}$, is calculated by the number of ACKs (i.e., 11 bytes long) that are received. The remaining time is spent idle. Therefore, the total energy spent is equal to: $E=V\cdot (i_{tx}\cdot t_{tx}+i_{rx}\cdot t_{rx}+i_{idle}\cdot t_{idle}+i_{sleep}\cdot t_{sleep})$. The current draw per mode is given in Table 5. Specifically, the current draw in the transmission mode, $i_{tx}$, is provided in the CC2420 datasheet for some transmission power levels, while the other values are evaluated through a quadratic fitting curve. In Table 5, the values of the current in the transmission mode are ordered accordingly to the list of power levels. The total energy consumption is equal to 1.78 Joule. Considering that TelosB nodes are supplied by 2 AA alkaline batteries, the capacity is twice 3000 mAh. Thus, the energy consumed by the transmitter over 750 min is 0.0027% of the available supply. In comparison, if we assume that the same amount of transmissions would have been executed by the highest transmission power level (i.e., 0 dBm), the energy consumption is 1.88 Joule, equal to 0.0029% of the energy supply.

## 7. Discussion

Our nodes are able to automatically adapt their transmission power to the varying conditions of the environment. The conditions have been changed per scenario by means of the distance between the transmitter and receiver of each node’s pair, the number of simultaneous transmitters and the traffic load. We have seen that increasing the value of the variables, the nodes raise their power. For each scenario, the QoS requirement of the system is the same: PRR higher than 95% using the minimum transmission power. The research question of this paper is about the transmission power that should be used to fulfill the QoS constraints, while minimizing the interference to the neighbours. Measurements and analysis per link at the network’s installation phase may provide useful hints, but then it is legitimate to ask ourselves whether users, or someone on their behalf, want to make measurements before using their products within the IoT market. Initial measurements would only be relevant for the static conditions tested at deployment time. However, the environment is influenced by changes (e.g., moving objects, new objects, variable number of people, variable number of sensor nodes), thus the power should be adaptive and autonomous (no user intervention). For instance, if the transmitter is moved further away from its recipient, it causes an increment on the transmitted signals’ attenuation and a drop on the received signal power. Hence, the transmission power needs to increase. Yet, if the number of transmitters in the network increases, a receiver will detect more interference. In both the cases, the receivers’ *SINR* worsens, affecting the QoS. The solution is either lowering the interferers power or boosting the transmitting signal. However, the limitation is that decreasing other nodes’ power influences their own network performance, whereas increasing one transmitter’s power produces more interference to the neighbours. There might be a trade-off that allows each node to satisfy their own performance constraints. A typical industrial solution is to set the transmission power to its maximum. This would be the easiest and apparently the most conservative approach, which would however consume energy excessively and waste spectrum, issues that become unsurmountable in high-density scenarios.

The benefit of our approach is that the nodes learn the minimum power they can use, without having to rely on any previous knowledge about the environment. Additionally, our self-learning process can take QoS requirements into account. Even though, in this paper, we have addressed only PRR, our method could consider any other features during the self-learning process, by modifying the nodes reward function. We could also set different goals for different nodes or subnetworks; there is no need to set up the learning process uniformly. In our work, we adopt the Manhattan grid topology, which is symmetric in the node disposition. Each transmitter in the multi-agent system has the same target, namely the QoS requirements. Therefore, as expected, the nodes learn similar transmission power levels, which are within a range smaller than 4 dBm. As future work, it would also be straightforward to study our protocol in asymmetric topologies, with dissimilar QoS constraints per node, whereby the transmission power varies across nodes.

## 8. Conclusion and future work

We have proposed a protocol that controls the effects that transmission power has on wireless sensor communications. The environment is formulated for the first time in WSNs as a Decentralized Partially Observable Markov Decision Process (Dec-POMDP), which is solved by an online reinforcement learning algorithm. Such formulation is realistic for a wireless network with a distributed architecture and composed by multiple nodes. Each node exploits its own local information of the environment in the algorithm. Such information is not transferred to other nodes, thus we avoid overheads in the network. The nodes are independent learners by observing the environment during packet transmissions, and players of a common interest theoretical game. The node’s cooperation is beneficial to the global network in terms of power reduction, such as to minimize the interference and prolong their battery lifetime.

Our TPC protocol is compared to the case of constant transmission power (i.e., homogeneous network). The results show that the system is adaptive to different scenarios, varying the interference and path loss, in a dynamic environment. The PRR is always higher than 95%, satisfying the requirement range 95–100%. The packet delay difference between the TPC protocol and the minimum value in the homogeneous case is lower than 14%. The maximum energy saving is 52.57% with respect to the homogeneous case. Finally, we have implemented the protocol in real sensor devices and shown the learning curve and performance improvement for one point-to-point communication.

Although our prototype and simulation work suggest great potential in self-controlled WSN based on learning, there are various ways in which our method may be further improved. First, we consider a deterministic time scheduling for the phases of learning and converging, followed by a testing period. It will be interesting to explore how time may be adjusted in face of different scenarios and conditions, for instance with different traffic patterns and traffic rates. We intend to study ways to consider together the scheduling time, the learning, discount and exploration factors with the action-value learning in time (i.e., the difference between two consecutive values is lower than a constant). Based on the stochastic analysis, the system switches between phases autonomously. In addition, the learning, converging and testing phases should take into account topology changes, such as either new nodes join the network or the existing nodes move or the environment changes (e.g. new obstacles, people activity). Such changes are represented by states that may have not been previously explored, thus the action-value is still equal to the initial value. A solution could be the use of transfer learning at the end of each phase, where the knowledge of the explored states is transferred to the unexplored ones [61]. In this way, convergence may get faster and the system would be able to adjust to a range of environmental conditions. Our protocol has been designed for real devices. Therefore, we have also presented an experimental work with sensor nodes that learn which transmission power to use in a real environment, aiming to maximize the PRR. It would be interesting to extend our work to carry out a more extensive comparative analysis and, in turn, address any deficiencies in the simulator.

## Figures and Tables

**Table 1:** Parameters and settings used in the simulator.

Parameter	Symbol	Value
Frequency channel	*f*	26
Distance of consecutive pair transmitters	*D*	2 m
Distance transmitter-receiver	*d*	2–4 m
Packets’ payload size	payload	50 bytes
Inter-arrival time	μ	25, 50, 75, 100 ms
Number of packets in a window	*N*	10
Number of transmission power levels	$n_{ptx}$	20
Minimum transmission power	$P_{tx,min}$	−35 dBm
Maximum transmission power	$P_{tx,max}$	10 dBm
Number of states	$n_{s}$	68
Reward quantization step size factor	Δ	5
Number of reward quantization levels	$m_{r}$	400
Number of PRR quantization levels	$m_{prr}$	20
Number of maximum retransmissions	$n_{retr}$	3
Number of maximum CCA attempts	$n_{CCA}$	4
Number of simulation runs	R	10

**Table 2:** Current specification of the AT86RF233 transceiver per radio status.

Radio status	Parameter	Value
TX_ON	$i_{tx}$	11.8 mA @ 0 dBm
RX_ON	$i_{rx}$	11.8 mA
SWITCH	$i_{switch}$	6 mA

**Table 3:** Scheduling of α, ϵ and γ over time.

Phase	ϵ	α	γ	Time (s)
Learning	1	0.9	0.8	t<600
	0.7	0.9	0.8	600≤t<1200
	0.3	0.9	0.8	1200≤t<1800
	0.1	0.9	0.8	1800≤t<2400
Convergence	0.1	0.1	0.8	2400≤t<3000
	0.1	0.01	0.8	3000≤t<3600
	0.1	0.001	0.8	3600≤t<4200
Testing	0	0.0001	0.8	4200≤t≤6000

**Table 4:** Quantization levels for CCA attempts, retransmissions and latency.

Quantization Level	*cca*	*retr*	*lat*
0	1	0	NA
1	2	1	low
2	3	2	medium
3	4	3	high

**Table 5:** Parameters and settings used in the experiments.

Parameter	Symbol	Value
Frequency channel	*f*	26
Distance transmitter-receiver	*d*	8 m
Packet’ payload size	payload	8 Byte
Inter-arrival time	μ	3 s
Number of packets in a window	*N*	10
Duty cycle	dc	38.4%
Number of transmission power levels	$n_{ptx}$	8
Transmission power	$P_{tx}$	0, −1, −3, −5, −7, −10, −15, −25 dBm
Current draw (transmit mode)	$i_{tx}$	17.4, 16.6, 15.2, 14, 12.8, 11, 9.9, 8.5 mA
Current draw (receiver mode)	$i_{rx}$	18.8 mA
Current draw (idle mode)	$i_{idle}$	20 μA
Current draw (sleep mode)	$i_{sleep}$	1 μA
Voltage supply	*V*	3 V
Number of states	$n_{s}$	64
Number of maximum retransmissions	$n_{retr}$	3
Number of maximum CCA attempts	$n_{CCA}$	4
Learning factor	α	0.8
Discount factor	γ	0.8

## References

[1] Freeman R.L. (2005). Fundamentals of Telecommunications.

[2] Santi P. (2005). Topology Control. Topology Control in Wireless Ad Hoc and Sensor Networks.

[3] Akyildiz I.F., Kasimoglu I.H. (2004). Wireless sensor and actor networks: Research challenges. Ad Hoc Netw..

[4] Ogundile O.O., Alfa A.S. (2017). A Survey on an Energy-Efficient and Energy-Balanced Routing Protocol for Wireless Sensor Networks. Sensors.

[5] Sendra S., Lloret J., Garcia M., Toledo J.F. (2011). Power Saving and Energy Optimization Techniques for Wireless Sensor Neworks. J. Commun..

[6] Jawad H., Nordin R., Gharghan S., Jawad A., Ismail M. (2017). Energy-Efficient Wireless Sensor Networks for Precision Agriculture: A Review. Sensors.

[7] Liotta A., Geelen D., van Kempen G., van Hoogstraten F. (2012). A survey on networks for smart–metering systems. Int. J. Pervasive Comp. Commun..

[8] Sheng Z., Yang S., Yu Y., Vasilakos A.V., Mccann J.A., Leung K.K. (2013). A survey on the ietf protocol suite for the internet of things: Standards, challenges, and opportunities. IEEE Wirel. Commun..

[9] Kotian R., Exarchakos G., Stavros S., Liotta A. (2017). Impact of Transmission Power Control in Multi-hop Networks. Futur. Gener. Comput. Syst..

[10] Liu T., Cerpa A.E. (2014). Temporal Adaptive Link Quality Prediction with Online Learning. ACM Trans. Sens. Netw..

[11] Liotta A. Farewell to Deterministic Networks. Proceedings of the 2012 IEEE 19th Symposium on Communications and Vehicular Technology in the Benelux.

[12] Chincoli M., Liotta A. (2018). Transmission Power Control in WSNs: From Deterministic to Cognitive Methods. Integration, Interconnection, and Interoperability of IoT Systems.

[13] Lin S., Zhang J., Zhou G., Gu L., Stankovic J.A., He T. (2006). ATPC: Adaptive transmission power control for wireless sensor networks. Proceedings of the 4th International Conference on Embedded Networked Sensor Systems.

[14] Jeong J., Culler D., Oh J.H. Empirical analysis of transmission power control algorithms for wireless sensor networks. Proceedings of the Fourth International Conference on IEEE Networked Sensing Systems.

[15] ElBatt T.A., Krishnamurthy S.V., Connors D., Dao S. Power management for throughput enhancement in wireless ad-hoc networks. Proceedings of the 2000 IEEE International Conference on Communications.

[16] Narayanaswamy S., Kawadia V., Sreenivas R.S., Kumar P. Power control in ad-hoc networks: Theory, architecture, algorithm and implementation of the COMPOW protocol. Proceedings of the European Wireless Conference.

[17] Fu Y., Sha M., Hackmann G., Lu C. Practical control of transmission power for wireless sensor networks. Proceedings of the 2012 20th IEEE International Conference on IEEE Network Protocols (ICNP).

[18] Kubisch M., Karl H., Wolisz A., Zhong L.C., Rabaey J. Distributed algorithms for transmission power control in wireless sensor networks. Proceedings of the 2003 IEEE Wireless Communications and Networking (WCNC 2003).

[19] Ikram W., Petersen S., Orten P., Thornhill N.F. (2014). Adaptive Multi-Channel Transmission Power Control for Industrial Wireless Instrumentation. IEEE Trans. Ind. Inform..

[20] Kim J., Kwon Y. (2009). Interference-aware topology control for low rate wireless personal area networks. IEEE Trans. Consum. Electron..

[21] Liotta A. (2013). The cognitive NET is coming. IEEE Spectr..

[22] Bosman H.H.W.J., Iacca G., Tejada A., Wörtche H.J., Liotta A. (2015). Ensembles of incremental learners to detect anomalies in ad hoc sensor networks. Ad Hoc Netw..

[23] Bosman H.H., Iacca G., Tejada A., Wörtche H.J., Liotta A. (2017). Spatial anomaly detection in sensor networks using neighborhood information. Inf. Fus..

[24] Kulkarni R.V., Forster A., Venayagamoorthy G.K. (2011). Computational Intelligence in Wireless Sensor Networks: A Survey. IEEE Commun. Surv. Tutor..

[25] Galzarano S., Liotta A., Fortino G. (2013). QL-MAC: A Q-learning based MAC for wireless sensor networks. Algorithms and Architectures for Parallel Processing.

[26] Yau K.L.A., Goh H.G., Chieng D., Kwong K.H. (2015). Application of reinforcement learning to wireless sensor networks: Models and algorithms. Computing.

[27] Galzarano S., Savaglio C., Liotta A., Fortino G. Gossiping-Based AODV for Wireless Sensor Networks. Proceedings of the 2013 IEEE International Conference on Systems, Man, and Cybernetics.

[28] Nitti M., Murroni M., Fadda M., Atzori L. (2016). Exploiting Social Internet of Things Features in Cognitive Radio. IEEE Access.

[29] Azizi R. (2016). Consumption of Energy and Routing Protocols in Wireless Sensor Network. Netw. Protoc. Algorithms.

[30] Chincoli M., Syed A.A., Exarchakos G., Liotta A. (2016). Power Control in Wireless Sensor Networks with Variable Interference. Mob. Inf. Syst..

[31] Liang X., Balasingham I., Leung V.C.M. Cooperative Communications with Relay Selection for QoS Provisioning in Wireless Sensor Networks. Proceedings of the 2009 IEEE Global Telecommunications Conference.

[32] Alsheikh M.A., Hoang D.T., Niyato D., Tan H.P., Lin S. (2015). Markov Decision Processes with Applications in Wireless Sensor Networks: A Survey. IEEE Commun. Surv. Tutor..

[33] Pandana C., Liu K. (2005). Near-optimal reinforcement learning framework for energy-aware sensor communications. IEEE J. Sel. Areas Commun..

[34] Gatsis K., Ribeiro A., Pappas G.J. (2014). Optimal Power Management in Wireless Control Systems. IEEE Trans. Autom. Control.

[35] Madan R., Lall S. (2006). An Energy-Optimal Algorithm for Neighbor Discovery in Wireless Sensor Networks. Mob. Netw. Appl..

[36] Stabellini L. Energy optimal neighbor discovery for single-radio single-channel wireless sensor networks. Proceedings of the 2008 IEEE International Symposium on Wireless Communication Systems.

[37] Lange S., Gabel T., Riedmiller M. (2012). Batch reinforcement learning. Reinforcement Learning.

[38] Sutton R.S., Barto A.G. (1998). Introduction to Reinforcement Learning.

[39] Krishnamurthy V., Ngo M.H. A game theoretical approach for transmission strategies in slotted ALOHA networks with multi-packet reception. Proceedings of the IEEE International Conference on Acoustics, Speech, and Signal Processing (ICASSP 2005).

[40] Udenze A., McDonald-Maier K. Partially Observable Markov Decision Process for Transmitter Power Control in Wireless Sensor Networks. Proceedings of the ECSIS Symposium on Bio-Inspired Learning and Intelligent Systems for Security (BLISS 2008).

[41] Kobbane A., Koulali M.A., Tembine H., Koutbi M.E., Ben-othman J. Dynamic power control with energy constraint for Multimedia Wireless Sensor Networks. Proceedings of the 2012 IEEE International Conference on Communications (ICC).

[42] Aprem A., Murthy C.R., Mehta N.B. (2013). Transmit Power Control Policies for Energy Harvesting Sensors With Retransmissions. IEEE J. Sel. Top. Signal Proc..

[43] Nourian M., Leong A.S., Dey S. (2014). Optimal Energy Allocation for Kalman Filtering Over Packet Dropping Links With Imperfect Acknowledgments and Energy Harvesting Constraints. IEEE Trans. Autom. Control.

[44] Yadav A., Goonewardena M., Ajib W., Dobre O.A., Elbiaze H. (2017). Energy Management for Energy Harvesting Wireless Sensors with Adaptive Retransmission. IEEE Trans. Commun..

[45] Liang X., Chen M., Leung V.C.M., Balasingham I. Soft QoS Provisioning for Wireless Sensor Networks: A cooperative communications approach. Proceedings of the 2010 5th International ICST Conference on Communications and Networking in China.

[46] Gummeson J., Ganesan D., Corner M.D., Shenoy P. (2010). An adaptive link layer for heterogeneous multi-radio mobile sensor networks. IEEE J. Sel. Areas Commun..

[47] Lin Z., van der Schaar M. (2011). Autonomic and Distributed Joint Routing and Power Control for Delay-Sensitive Applications in Multi-Hop Wireless Networks. IEEE Trans.Wirel. Commun..

[48] Udenze A., McDonald-Maier K. Direct Reinforcement Learning for Autonomous Power Configuration and Control in Wireless Networks. Proceedings of the NASA/ESA Conference on Adaptive Hardware and Systems, AHS 2009.

[49] Le T.T.T., Moh S. (2017). An Energy-Efficient Topology Control Algorithm Based on Reinforcement Learning for Wireless Sensor Networks. Int. J. Control Autom..

[50] Sung Y., Ahn E., Cho K. (2013). Q-learning Reward Propagation Method for Reducing the Transmission Power of Sensor Nodes in Wireless Sensor Networks. Wirel. Pers. Commun..

[51] Kazemi R., Vesilo R., Dutkiewicz E., Liu R. Dynamic power control in Wireless Body Area Networks using reinforcement learning with approximation. Proceedings of the 2011 IEEE 22nd International Symposium on Personal, Indoor and Mobile Radio Communications.

[52] Hu J., Wellman M.P. (2003). Nash Q-learning for general-sum stochastic games. J. Mach. Learn. Res..

[53] Wiering M., van Otterlo M. (2012). Reinforcement Learning. Adaptation, Learning, and Optimization.

[54] Chincoli M., Syed A.A., Mocanu D.C., Liotta A. Predictive Power Control in Wireless Sensor Networks. Proceedings of the 2016 IEEE First International Conference on Internet-of-Things Design and Implementation (IoTDI).

[55] IEEE Computer Society, LAN/MAN Standards Committee, Institute of Electrical and Electronics Engineers, IEEE-SA Standards Board (2006). IEEE Standard for Information Technology Telecommunications and Information Exchange between Systems–Local and Metropolitan area Networks–Specific Requirements.

[56] Series P. (2009). Propagation Data and Prediction Methods for the Planning of Indoor Radiocommunication Systems and Radio Local Area Networks in the Frequency Range 900 MHz to 100 GHz.

[57] Nakagami M., Hoffmann W. (1960). The m-distribution – A general formula of intensity distribution of rapid fading. Statistical Methods in Radio Wave Propagation.

[58] Atmel 8351 MCU Wireless AT86RF233 Datasheet. http://www.atmel.com/images/Atmel-8351-MCU_Wireless-AT86RF233_Datasheet.pdf.

[59] Dunkels A. (2011). The contikimac radio duty cycling protocol.

[60] CC2420 Single-Chip 2.4 GHz IEEE 802.15.4 Compliant and ZigBee^TM^ Ready RF Transceiver. http://www.ti.com/product/CC2420.

[61] Torrey L., Shavlik J. (2009). Transfer learning. Handbook of Research on Machine Learning Applications and Trends: Algorithms, Methods, and Techniques.

